# Implementing and Evaluating Face-to-Face Near-Peer Teaching in Response to the Absence of Objective Structured Clinical Examinations (OSCEs) for Junior Medical Students Following the COVID-19 Pandemic

**DOI:** 10.7759/cureus.74540

**Published:** 2024-11-26

**Authors:** Sheryll N Kamat, Rishil A Patel, Ria Patel

**Affiliations:** 1 Internal Medicine, University College Hospital, London, GBR; 2 Internal Medicine, Chesterfield Royal Hospital, London, GBR

**Keywords:** clinical competencies, clinical skills, covid 19, graduate outcomes, medical education, near peer teaching, objective structured clinical examination (osce), objective structured clinical examinations (osces), objective structured clinical exam (osce), simulated clinical environment

## Abstract

Introduction

The COVID-19 pandemic resulted in the suspension of formative Objective Structured Clinical Examinations (OSCEs) at numerous institutions. This resulted in a significant gap in OSCE exposure for junior medical students, including those at our university. Drawing upon our prior experiences with OSCEs, we created a program to evaluate the effectiveness of near-peer teaching (NPT) in preparing third-year medical students to experience and succeed in OSCEs. This program was conducted at the University of Buckingham Medical School (Milton Keynes, GBR) from July to October 2022.

Methodology

Two final-year medical students, under a medical consultant's supervision, led the program development and implementation. This consisted of 2.5 hours of weekly face-to-face OSCE simulation over 12 weeks. The program was designed to mirror the university's OSCE format but provided the intended benefit of designated feedback and teaching at the end of each station. Each week, five dedicated final-year medical students constructed OSCE stations focused on common clinical presentations and skills. Sixty third-year medical students who had completely missed formative OSCEs due to the COVID-19 pandemic enrolled in the program. Pre- and post-session surveys were administered to assess the effectiveness of the NPT program among students.

Results

In our study, the paired t-test analysis of data showed a statistically significant improvement after attending our OSCE NPT program in all three domains assessed. Participants reported enhanced preparedness in OSCE (p<0.001), a better awareness of the OSCE structure (p<0.001), and an improved understanding of how General Medical Council (GMC) 'Outcomes for Graduates' themes may be assessed (p<0.001). These findings collectively suggest that the NPT intervention effectively enhanced the participants’ clinical competencies and preparedness for OSCEs.

Conclusion

The peer-led OSCE preparation session, facilitated by senior medical students, effectively augmented third-year medical students' clinical skills and knowledge, enhancing their readiness for the OSCE. These sessions provided a unique opportunity for skill development and practice in a simulated clinical environment, fostering greater confidence and proficiency among participating students. Our findings highlight the crucial role of NPT in preparing students for OSCEs. We recommend using NPT to supplement learning, both during periods when traditional OSCEs are unavailable and as a regular adjunct to medical education.

## Introduction

The Objective Structured Clinical Examination (OSCE) is a standardized assessment that plays a pivotal role in medical education [1]. Its widespread use in medical schools globally provides a methodology to evaluate the clinical competence of medical students preparing to become future doctors. The OSCEs are designed to comprehensively assess both the theory and practical application of skills in a controlled, simulated environment. Direct observation of performance allows students to showcase their problem-solving, situational judgment, and interpersonal skills; proficiencies that are traditionally challenging to assess. Thus, OSCEs reassure medical educators that students are exposed to the necessary competencies to practice safely and effectively [2]. 

The COVID-19 pandemic has profoundly and unprecedentedly disrupted the traditional course of medical education [3]. In March 2020, the United Kingdom implemented a nationwide lockdown in response to the COVID-19 pandemic. This resulted in the widespread suspension of face-to-face formative examinations in many universities, preventing third-year medical students at our institution and elsewhere from experiencing OSCEs. The mandatorily imposed isolation restrictions led to the abrupt closure of clinical placements, forcing a comprehensive transition from in-person to online learning environments. This had far-reaching implications for the acquisition of clinical skills and knowledge by medical students, particularly those traditionally assessed through OSCEs [4]. Post-pandemic, the relaxation of national lockdown restrictions enabled a return to in-person learning and face-to-face OSCEs. However, for the medical cohort 2020 at our institution, the shift to remote assessments during the pandemic led to a significant lack of exposure to both preparation for and experience with face-to-face OSCEs. 

Near-peer teaching (NPT) is an educational strategy in which students, typically one or more academic years ahead of their peers, take on a teaching role. In the context of medical education, this involves senior medical students educating junior colleagues on various medical topics or skills. This strategy offers unique benefits for both tutors and tutees, particularly in skill-based learning such as clinical procedures and communication skills [5]. For senior students, this process helps them with supplemental learning and develops their teaching, leadership, teamwork, and communication skills. For junior students, peer-to-peer interaction fosters a collaborative and supportive learning environment. Students share similar experiences and perspectives, enhancing communication and understanding [6]. 

Leveraging on our OSCE experience, we conducted a study to evaluate the efficacy of NPT in preparing third-year medical students for their formative OSCEs. This involved the development and implementation of the OSCE-NPT program. A 12-week program designed by final-year medical students for their junior peers, consisting of 2.5 hours of weekly OSCE simulation and teaching. 

## Materials and methods

Program development and design 

A 12-week NPT method curriculum was designed to augment traditional learning methods and prepare third-year medical students for the formative OSCEs. The NPT approach was selected due to its known effectiveness in fostering a supportive and collaborative learning environment [6]. By leveraging the knowledge and insights of more experienced peers, junior students could benefit from relatability, guidance, and immediate feedback, ultimately enhancing their learning experience. We requested students to complete a pre-and post-session questionnaire. This data was then analyzed using paired t-tests to determine the effect of NPT teaching in preparation for OSCEs. 

The program had four objectives: familiarize students with the OSCE format, structure, and timing; enhance students' confidence in approaching OSCE stations; develop students’ clinical skills and communication abilities through simulated patient encounters; and provide immediate, specific feedback and teaching on performance through the NPT model. Each week, the senior medical student mentors developed stations focusing on common clinical presentations and skills. We utilized a shared Google document (Google LLC, Mountain View, CA, USA) to ensure that station content was original and of high quality each week. The mentor team ensured all stations were meticulously constructed, with an emphasis on meeting the standards of patient-centered care as set by the General Medical Council's (GMC) ‘Outcomes for Graduates’ framework [7]. Skills that were examined at each station included history taking, examinations, communication skills, interpretation of investigations, professional knowledge, and procedural skills using mannequins. For procedural skills, we liaised with the university's clinical skills team to access equipment. They also provided us with the rubrics for these procedures, in line with the expectations of our curriculum. Before each session, the five mentors provided detailed scripts to student actors outlining case scenarios, expected patient behavior, and appropriate responses to student queries. This standardized approach ensured consistent and realistic portrayals across all stations.

We used the ‘Pendleton Method of Giving Feedback’ [8] to allow us to recognize qualities worth commending and stimulate self-reflection among participants. The mentors developed formal mark schemes for each station, which were shared with the third-year medical students post-session. These rubrics outlined key assessment criteria and provided recommended guidance and resources for further learning relating to the station.

Recruitment 

Third-year medical students were invited to participate voluntarily in the program. Recruitment strategies included email, broadcast communications, and distributing informational posters around high-traffic areas of the medical school campus. Students who expressed an interest in participation were invited to join a WhatsApp (Meta Platforms Inc., Menlo Park, CA, USA) group where weekly session details (date, location, and time) were communicated.

To maximize the number of participants while preserving program quality, a sample size calculation was performed. Considering the program's requirements for dedicated faculty and resources, a maximum of five students per weekly session was identified as being feasible. We determined five stations to be optimal in providing sufficient variety and exposure to the OSCE format while allowing adequate feedback time.

Initially, we considered a smaller cohort of students who would participate in multiple sessions. However, to maximize the number of students exposed to the simulation, we opted to enroll the entire eligible cohort, ensuring at least one opportunity for each participant. Sixty third-year medical students were enrolled in our program, all of whom had been unable to participate in any formative OSCEs due to the pandemic-induced lockdown.

Five senior medical students served as program mentors. They were selected for their passion for medical education and longitudinal learning, demonstrating the requisite qualities for effective teaching. All mentors had previous experience delivering teaching sessions in programs run by medical school societies. They all demonstrated exceptional performance in their formative OSCEs, indicating valuable knowledge and understanding of the OSCE format. Additionally, five final-year medical students were recruited as standardized patients to enhance the authenticity of the simulated OSCE environment. This offered a cost-effective and practical approach. 

Program structure

The OSCE program was conducted at the University of Buckingham Medical School (Milton Keynes, GBR) from July to October 2022. It involved a weekly session of 2.5 hours, delivered over 12 weeks (Table 1). Each week, a group of five junior medical students participated in a single session consisting of five uniquely designed OSCE stations, which they rotated through in a circuit. This process was repeated weekly, each time with a new batch of five students, thereby ensuring all 60 students got to participate in the program. The delivery of these sessions aimed to replicate the structure and timing of our university’s OSCEs. Although we allocated two hours, feedback and teaching times often ran over. Additional time was also needed for procedural stations that required the examiner to reset the stations. In total, sessions typically took around 2.5 hours. Upon completing the program, all station briefs from the 12-week program were shared with all 60 junior medical students, along with guidance and resources for each station. This allowed them to revise and practice collaboratively, ensuring equal access to the program's content, topics, and themes.

**Table 1:** Key components of the OSCE program structure OSCE: Objective Structured Clinical Examination, NPT: Near-peer teaching

OSCE program components	Details
Total number of students enrolled in the program	60 students
Total number of weeks	12 week
Weekly session duration	2.5 hours
Number of stations per session	Five stations
Per station participants	One junior medical student, up to two senior medical students (one NPT mentor and one standardized patient as required)
Meet and greet at the start of each weekly session	Five minutes (involving all student mentors, actors, and participants)
Station circuit	1. Allocated station-brief reading time (one minute); 2. Station activity (10 minutes); 3. Station debrief and teaching (10 minutes); 4. Rotate to the next station and repeat steps one to four until each student has completed all five stations
Circuit completion	Conclusion, the opportunity for final questions, and request for a post-session survey.

Feedback 

Each week we administered anonymous pre- and post-session surveys to junior students and a final survey to senior medical students upon completion of the program. We used the 5-point Likert scale, dichotomous (yes/no) questions, and free-text responses to collect quantitative and qualitative data. Our pre-session survey for junior students (see Appendix A) evaluated students' perceived preparedness for their upcoming OSCEs, awareness of the OSCE structure, understanding of how relevant 'GMC outcomes for graduates' themes would be assessed, and the specific impacts that the pandemic had on their preparedness for OSCEs. Our post-session survey (see Appendix B) evaluated the changes in students’ perceived preparedness for upcoming OSCEs, awareness of the OSCE structure, and understanding of how relevant 'GMC outcomes for graduates' themes would be assessed. Additionally, we evaluated specific aspects of the session that students found most effective or beneficial to their learning, suggested areas for improvement, the relevance of content in line with the university curriculum, and the organization of the session. Individualized teaching feedback for mentors was requested separately. A post-program survey for senior medical students (see Appendix C) evaluated the benefits of NPT in enhancing their learning through the development of teaching, leadership, teamwork, and communication skills. We also explored teachers’ perceived benefits of the NPT program for our junior peers. 

## Results

Results of the pre- and post-session survey for junior medical students 

**Figure 1:**
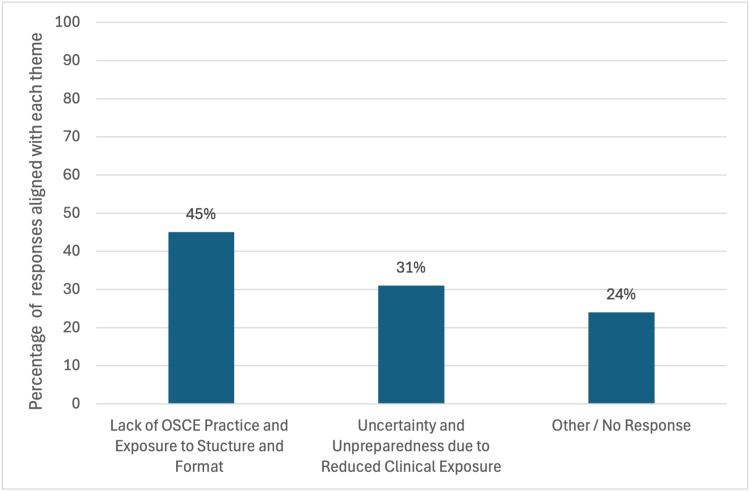
Key themes in student responses to the question 'What effect did the COVID-19 pandemic have on your preparedness for clinical exams?' OSCE: Objective Structured Clinical Examination

Of the 60 students enrolled, 56 (93%) completed pre- and post-session surveys. Ninety-five percent of students who completed the pre-session survey reported that the COVID-19 pandemic significantly affected their preparedness for OSCEs. Further analysis of qualitative responses provided insights into the specific effects of this. These could be categorized into three key themes: lack of OSCE practice and exposure to structure/format, unpreparedness due to reduced clinical exposure, and other or no response. Figure 1 shows the percentage distribution of the three identified themes, illustrating their relative prevalence among student responses. Table 2 shows representative quotes demonstrating themes. 

**Table 2:** Key themes in student responses to the question 'What effect did the COVID-19 pandemic have on your preparedness for clinical exams?' OSCE: Objective Structured Clinical Examination

Lack of OSCE practice and exposure to structure/format	Unpreparedness due to reduced clinical exposure
‘Less understanding of OSCE assessment structure, expectations and marking’	‘Less exposure to clinical teaching. Fewer opportunities to practice examinations on real patients.’
‘It took away the opportunity to enhance examination skills, it didn’t give us the opportunity to go through an OSCE and understand what it entails and how questions will be asked’	‘I feel there’s a knowledge gap due to online teaching which cannot replace face-to-face practical learning’
‘Without the pressure and intensity of real exams, there is no way to be properly prepared for an assessment of this nature’	‘I learn best through hands on experience with patients and real examinations, which wasn’t possible online’
‘Reduced frequency of OSCE practice hence reduced fluidity in clinical skills’	‘Lack of face-to-face clinical skills and communication in placement’
‘No experience with previous OSCE format or experience preparing for these’	‘Unable to learn and refine exam techniques’

We analyzed our pre- and post-survey Likert scale data using paired t-tests to determine whether there was an improvement in perceived OSCE preparedness following the NPT program. The analysis revealed highly significant differences between pre-test and post-test scores in all domains (p<0.001) demonstrating the statistical significance of our results (Table 3).

**Table 3:** Paired t-test analysis of pre- and post-Likert scale data OSCE: Objective Structured Clinical Examination, NPT: Near-peer teaching, GMC: General Medical Council

Survey statements	Pre-session survey mean	Post-session survey mean	Pre-session survey standard deviation	Post-session survey standard deviation	Two-tailed p-value
I feel prepared to sit for my upcoming OSCE	2.6	4.6	1.0	0.9	<0.001
I am aware of how the OSCE will be structured	3.6	4.8	1.1	0.8	<0.001
I understand how the relevant 'GMC outcomes for graduates' themes will be assessed	2.8	4.4	0.9	0.9	<0.001

**Figure 2:**
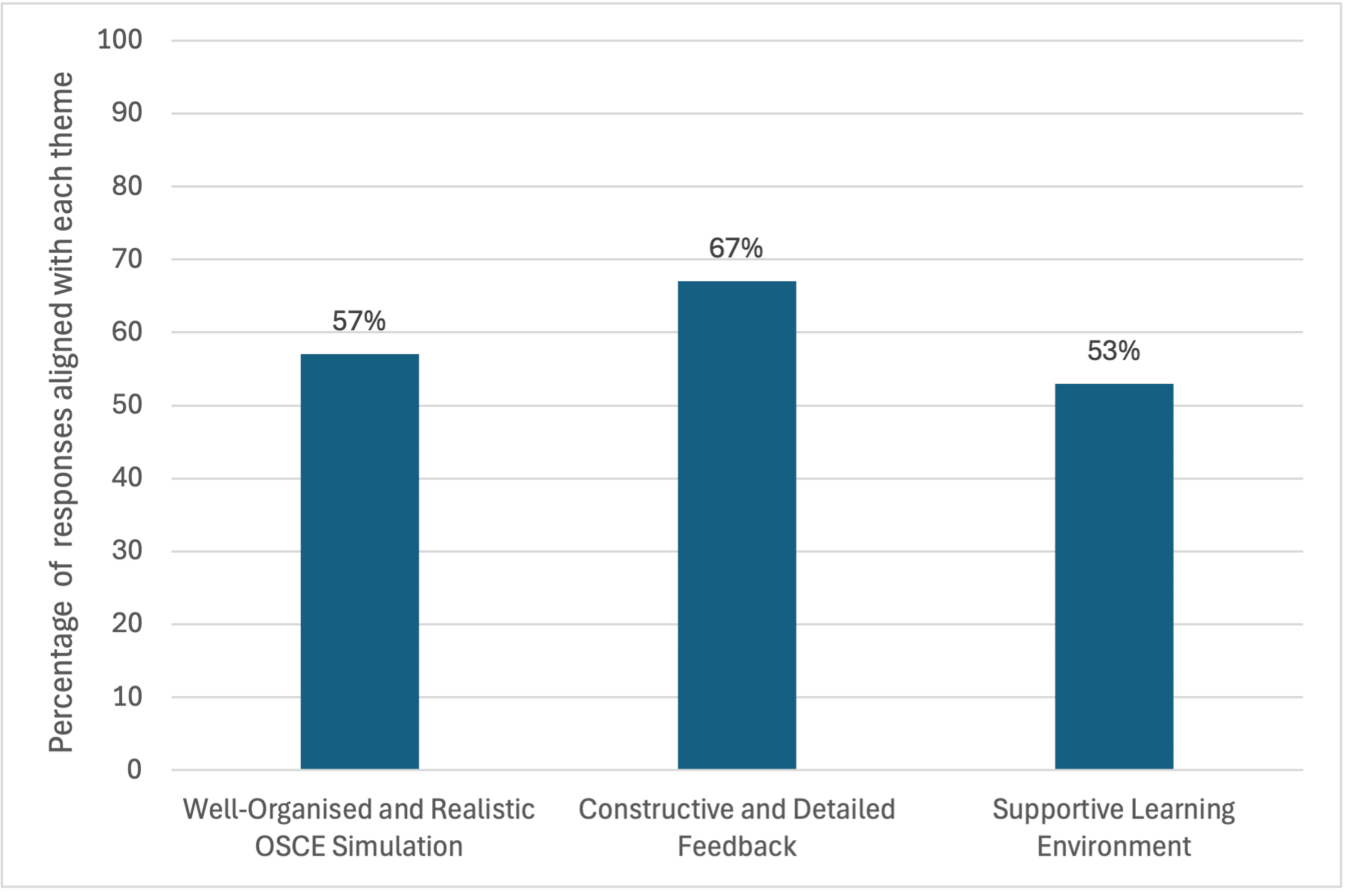
Key themes in student responses to the question 'Which aspects of the program were most effective or beneficial?’ OSCE: Objective Structured Clinical Examination

Ninety-six percent of students 'strongly agreed' or 'agreed' that the sessions were well organized and relevant to our university curriculum. Our post-session survey explored aspects of the program that were most effective or beneficial to their learning. Feedback was analyzed and divided into three key themes: well-organized and realistic OSCE simulation, constructive and detailed feedback, and a supportive learning environment. There was a notable overlap of responses; hence, these were categorized within more than one theme to capture the nuanced perspectives of students. Themes are represented as percentages, as shown in Figure 2. Representative quotes demonstrating themes are shown in Table 4. 

**Table 4:** Representative quotes illustrate key themes in student responses to the question, ‘Which aspects of the program were most effective or beneficial to your learning?’ OSCE: Objective Structured Clinical Examination

Well-organized and realistic OSCE simulation	Constructive and detailed feedback	Supportive learning environment
‘Really well organised. I definitely have a better understanding of the structure and what to expect going into the real OSCE.’	‘The excellent and very individualized feedback, every *examiner* and *actors* were friendly; very useful tips to think about for future practices.’	‘Everyone was friendly and approachable. I didn’t feel scared to ask questions or make mistakes.'
‘It was so well organized and well structured. Very relevant.’	‘Feedback was extremely useful. Very helpful teaching and tips on areas of improvement.’	‘Fun, and I felt like I was learning! I went in with limited knowledge but everyone was so kind and did not make me feel bad for not knowing.'
‘Enjoyed the accurate representation of what an OSCE is actually like’	‘Really thorough feedback on performance was given so now I know what to work on’	‘Everyone was very helpful and happy to answer questions. I honestly learnt so much.'
‘Stations were well put together and the use of actors made it very realistic. The content was extremely relevant.’	‘Amazing explanations and feedback on how to improve and what to do differently'	‘I felt so supported and encouraged the entire time. This really helped build confidence in my own abilities.’
‘Good mixture of different types of stations and the content was varied too. Very realistic.’	‘The feedback was very accurate, clear, and relevant.’	‘Teachers and actors were so helpful, made the learning experience very enjoyable’

Feedback on areas of improvement was largely favourable with a majority of students indicating that no changes were necessary. Specific individual suggestions included longer time for teaching and sessions facilitating 20-minute stations. One hundred percent of students would recommend this program to their peers.

Results of the post-session survey for student mentors

All five mentors completed the end-of-program survey. One hundred percent of the mentors 'strongly agreed' that the program was beneficial to their learning and allowed the development of leadership, teamwork, and communication skills. Representative quotes organized into the aforementioned domains alongside perceived benefits to junior students are shown in Table 5.

**Table 5:** Representative quotes illustrating qualitative mentor feedback on the benefits of NPT NPT: Near-peer teaching

Themes	Representative quotes
Leadership	‘The program empowered me to take initiative and lead my peers effectively. I developed key skills in fostering a collaborative team environment.’
Teamwork	‘Working alongside my colleagues involved sharing ideas, collaboration, and teaching and learning from each other.’
Communication	‘Understanding how my junior peers learn has allowed me to adapt my communication style to meet their needs. I have learned to ask questions to help them articulate their thoughts and have open discussions to facilitate learning.’
Perceived benefits to junior students	‘Students truly benefited and were grateful for tips and clinically relevant advice they received.’

## Discussion

The COVID-19 lockdown forced an abrupt and unprecedented change to medical education worldwide. Various institutions experimented with innovative online strategies to ensure continuity with curriculum education. While this was a good aid for academic learning, the practical aspects could not be replicated entirely in a remote environment [9]. In conversations with our junior peers, many expressed how they felt ‘unprepared’ and ‘anxious’ about OSCEs, specifically due to the impact of missed clinical exposure, practical skills, and formal assessment of these competencies. It is well documented that students find OSCEs stressful due to the pressure of performing well and concerns about the subjective nature of grading [10]. However, we were unable to find studies that have evaluated the effects of lockdown on students returning to a clinical environment, especially related to their OSCE preparedness and/or performance. 

Near-peer teaching has a longstanding established relationship within medical education. It is becoming increasingly recognized as an effective way to enhance learning [11]. Several studies highlight the value to both medical student tutors and tutees, including consolidation of knowledge, development of teaching skills, and social congruence [12-14]. Additionally, a comprehensive systematic review and meta-analysis concluded that NPT is likely the most effective method for improving procedural skills among students [15]. While medical faculty are essential for delivering the medical curriculum to students, voluntary near-peer teachers can offer a distinctive perspective and provide learners with a less intimidating environment for learning compared to other methods of teaching [14]. Given these promising findings, we implemented and evaluated NPT as an educational tool for OSCE preparation immediately following the lifting of the nationwide COVID-19 lockdown. 

Upon completing our program, participants reported a significant increase in their preparedness for their upcoming OSCEs. The findings reaffirm the research of Braier-Lorimer and Warren-Miell [16] and de Menezes and Premnath [14] who also observed that peer-led mock OSCEs enhanced student confidence for formative OSCEs. Our study is the first to report student perceptions on an understanding of how 'GMC outcomes for graduates' themes are assessed. Specifically, both NPT tutors and tutees unanimously reported that our program improved their communication and interpersonal skills [7]. These findings align with research highlighting the benefits of NPT and emphasizing communication as a key transferable skill in professional practice [11]. 

We attribute our success to several key factors. We set clear learning objectives that were explicitly defined and aligned with the OSCE assessment criteria. All learning activities were well structured. Our experience in conducting simulated OSCE scenarios, coupled with peer feedback and case discussions, fostered enhanced learning and retention. By providing immediate feedback and assessment directly after each station, we helped participants identify their strengths and weaknesses, enabling them to make necessary adjustments. Particularly in the context of OSCE, we found that the utilization of NPT created a uniquely effective learning experience for all participants. Senior medical students who had recently undergone OSCEs offered invaluable guidance to junior students. Our firsthand experiences, tips, and strategies provided a highly relevant and authentic perspective on the assessment process. This fostered a collaborative and supportive learning environment. Junior students felt more comfortable and less stressed asking questions, seeking feedback, and discussing their concerns with peers who had recently faced similar challenges. All students related easily to their mentor peers, leading to increased engagement and active participation. 

A post-program survey completed by mentors revealed a general satisfaction with the experience and the effectiveness of the NPT OSCE program. All mentors emphasized the program's contribution to their professional development in line with key themes set by the GMC's ‘Outcomes for Graduates’ framework [7]. They highlighted teamwork through collaboration and support of peers, development as teachers through enhanced communication and interpersonal skills and opportunity for further learning and consolidation of knowledge. Mentors reported that the NPT methodology created a supportive environment for learning and allowed students to ‘practice without fear of failure.' They agreed that the program familiarized students with the OSCE format and structure. Ultimately, the teachers observed a perceived increase in student confidence and preparedness for the OSCEs.

Study limitations

Although we had positive results, our study had several limitations. Our sample size of 60 students and enrollment based on a first-come, first-served basis is not accurately representative of the medical student population of that year. All data was collected through self-administered questionnaires completed immediately pre- and post-session. This approach may introduce favorable bias. Although a medical consultant oversaw our program, individual sessions were not generally attended. This posed the risk of knowledge limitations among student mentors. To mitigate this, we ensured mentors acknowledged the uncertainty and directed junior peers to seek clarification from specialty consultants. Additionally, we sought rubrics and mentor teaching for procedural skills to align with our curriculum. This could be further strengthened through an active re-evaluation of the process by accredited academic staff [17]. Our primary objective was to improve familiarity, preparedness, and confidence among junior peers. Our study could be improved by placing an equal emphasis on the learning and development of our mentors. For example, evaluation of weekly teaching objectives and facilitating training sessions for our mentors. Doing so would allow further development as clinical teachers and maximize the benefits to near-peer mentors.

## Conclusions

We successfully implemented and evaluated NPT as an education tool for junior medical students in response to the absence of OSCEs following the COVID-19 pandemic. Our study demonstrated mutual benefits for both mentors and students. Learners demonstrated increased preparedness for upcoming OSCEs. Teachers developed improved knowledge and transferable skills in teaching, leadership, teamwork, and communication that can be integrated into their future medical careers.

Near-peer teaching was found to be a cost-effective approach to OSCE preparation, requiring minimal additional resources and easily adaptable to various learning environments. We acknowledge the importance of ongoing evaluation of the NPT program and understand this cannot replace current evidence-based teaching such as clinical placements and designed simulation and teaching. We have since established our program as an ongoing adjunct to hands-on learning in our medical school. This has provided the opportunity for our junior students to become teachers for the upcoming year. We conclude that NPT is a valuable tool that can be readily customized to meet the specific needs of individual students or groups. It effectively complements traditional teaching methods and provides targeted support. 

## Appendices

Appendix A

Pre-Session Survey for Junior Medical Students

Please answer the following questions regarding your thoughts and feelings about your upcoming OSCE. Rate on the Likert scale: 0 = strongly disagree, 5 = strongly agree

1) I feel prepared for my upcoming OSCE

 2) I am aware of how the OSCE will be structured (rotations and content)

 3) I understand how relevant ‘GMC outcomes for graduates’ themes may be assessed 

Please answer the following question regarding the COVID-19 pandemic with a yes/no

4) The COVID-19 pandemic significantly affected my preparedness for upcoming OSCEs

5) If yes, how did the COVID-19 pandemic affect your preparedness for OSCE?

Appendix B

Post-Session Survey for Junior Medical Students

Please answer the following questions regarding your thoughts and feelings about your upcoming OSCEs following our OSCE program. Rate on the Likert scale: 0 = strongly disagree, 5 = strongly agree

1) I feel prepared for my upcoming OSCEs

2) I am aware of how the OSCEs will be structured (rotations and content)

3) I understand how relevant ‘GMC outcomes for graduates’ themes may be assessed 

4) The session was relevant to the university curriculum 

5) The session was well-organized 

6) What aspects of the session were effective or beneficial to your learning? (Free text response)

7) What could be done to improve these sessions? (Fre text response)

8) I would recommend this session to fellow medical students: Yes/No

Appendix C

Post-Program Survey for Senior Medical Students

To what extent do you agree that the OSCE program helped develop your skills in the following areas? Rate on the Likert scale: 0 = strongly disagree, 5 = strongly agree

1) Improved my own learning

2) Development of leadership skills 

3) Enhanced my teamwork skills

4) Improved my communication skills

5) Please comment on how the program addressed these areas. (Free text response)

6) How do you feel the program benefited the junior medical students? (Free text response)
